# Panton–Valentine Leukocidin Enhances the Severity of Community-Associated Methicillin-Resistant *Staphylococcus aureus* Rabbit Osteomyelitis

**DOI:** 10.1371/journal.pone.0007204

**Published:** 2009-09-25

**Authors:** Anne-Claude Crémieux, Oana Dumitrescu, Gerard Lina, Christian Vallee, Jean-François Côté, Martine Muffat-Joly, Thomas Lilin, Jerome Etienne, François Vandenesch, Azzam Saleh-Mghir

**Affiliations:** 1 Département de médecine aigüe, Hôpital Universitaire Raymond Poincaré, Assistance Publique-Hôpitaux de Paris, Garches, France; 2 EA 3647, Faculté de Médecine Paris-Ile-de–France Ouest, Université Versailles Saint Quentin, Versailles, France; 3 Inserm U851, Centre National de référence des Staphylocoques, Faculté Laennec, Université Lyon 1, Lyon, France; 4 Service de radiologie, Hôpital Universitaire Raymond Poincaré, Assistance Publique-Hôpitaux de Paris, Garches, France; 5 Service Anatomie Pathologique, Hôpital Ambroise Paré, Boulogne-Billancourt, France; 6 Inserm IFR2-Centre d'Explorations Fonctionnelles Intégré, UFR de Médecine Paris 7, site Bichat, Paris, France; 7 Centre de Recherches Biomédicales, Ecole Nationale Vétérinaire d'Alfort, Maisons Alfort, France; Columbia University, United States of America

## Abstract

**Background:**

Extensive spread of community-associated methicillin-resistant *Staphylococcus aureus* (CA-MRSA) in the United States, and the concomitant increase in severe invasive staphylococcal infections, including osteomyelitis, in healthy children, has led to renewed interest in Panton-Valentine leukocidin (PVL). However, the pathogenetic role of PVL in staphylococcal infections remains controversial, possibly because it depends on the site of infection.

**Methodology/Principal Findings:**

We compared the course of experimental rabbit osteomyelitis due to the PVL-positive CA-MRSA strain USA 300 (LAC) and its PVL-negative isogenic derivative (LACΔ*pvl*), using a low and a high inoculum (8×10^5^ and 4×10^8^ CFU). With the low inoculum, bone infection was less frequent on day 7 (D7) and day 28 (D28) with LACΔ*pvl* than with LAC (respectively 12/19 and 18/19 animals, p = 0.042). With the high inoculum of both strains, all the animals were infected on D7 and the infection persisted on D28 in almost every case. However, tibial bacterial counts and the serum CRP concentration fell significantly between D7 and D28 with LACΔ*pvl* but not with LAC. Respectively 67% and 60% of LAC-infected rabbits had bone deformation and muscle/joint involvement on D7, compared to 0% and 7% of LACΔ*pvl*-infected rabbits (p = 0.001 and p = 0.005 respectively). Between D0 and D28, the anti-PVL antibody titer increased significantly only with the high inoculum of LAC.

**Conclusions/Significance:**

PVL appears to play a role in the persistence and rapid local extension of rabbit osteomyelitis, in keeping with the greater severity of human bone infections due to PVL-positive *S. aureus*. The possible therapeutic implications of these findings are discussed.

## Introduction

Panton-Valentine leukocidin (PVL) is a hetero-oligomeric pore-forming toxin composed of two components (LukS-PV and LukF-PV) and produced by *Staphylococcus aureus*. Its prevalence in clinical *S. aureus* isolates used to be low but is currently increasing due to the worldwide spread of PVL-producing community-associated methicillin-resistant *S. aureus* (CA-MRSA) strains [1]. Regardless of methicillin resistance, PVL is particularly prevalent in staphylococcal strains that cause deep skin and soft-tissue infections, severe necrotizing pneumonia and severe bone and joint infections, all of which mainly affect children and young adults [2]–[5].

Extensive spread of CA-MRSA in the United States, mainly due to the exceptionally infectious strain USA300 [6], and the concomitant increase in severe invasive staphylococcal infections, including osteomyelitis, in healthy children [7]–[9], has renewed interest in the pathogenic role of PVL. Studies using various experimental models [10]–[13] have given conflicting results, however, raising the possibility that the role of PVL might depend on the site of infection, as well as the experimental model [14].

Osteomyelitis has long been recognized as a major clinical syndrome of invasive *S. aureus* disease [15], accounting for 7% of staphylococcal infections among children hospitalized in the United States [9]. A role of PVL in bone and joint infections was initially suspected by Panton and Valentine [16] and has recently been the focus of several studies, mostly in the pediatric setting. In a retrospective study, Martinez-Aguilar et al. [17] noted that musculoskeletal infection due to PVL-positive community-acquired (CA) MRSA seemed to be associated with more fever, longer hospitalization, and more local complications. In a prospective study comparing pediatric cases of osteomyelitis caused by PVL-positive *S. aureus* and PVL-negative *S. aureus*, Bocchini et al. found that patients with PVL-positive strains had significantly stronger inflammatory responses, more local complications such as myositis and pyomyositis, and more frequently positive blood culture [18]. Independently, Dohin et al compared pediatric cases of bone and joint infection caused by PVL-positive and PVL-negative *S. aureus* and confirmed that PVL-positive cases tended to be more severe and to require longer treatment; in addition, local complications were more frequent and often necessitated repeated surgical drainage [5].

Several experimental models, using mainly mice but also rabbits, have been developed in recent years to investigate the pathogenetic role of PVL, in necrotizing pneumonia, skin infections, and sepsis [10]–[12], [19], and also to test a PVL vaccine [19]. However, there have been no experimental studies of PVL in bone and joint infections.

The purpose of this study was to compare the virulence of the PVL-positive strain USA300 and its isogenic *pvl-*negative derivative in a rabbit model of acute osteomyelitis. Rabbits were chosen because their leukocytes are as susceptible as human polymorphonuclear neutrophils to PVL [20]. Strain USA300 was chosen because it is the most prevalent PVL-positive CA-MRSA strain in the United States.

Our results clearly showed that PVL plays a role in the aggressive course of experimental CA-MRSA osteomyelitis, a result highly consistent with previous clinical observations.

## Methods

### Bacterial strains

We used a clinical *S. aureus* strain belonging to the USA300 lineage, and its isogenic Δ*luk*S/F-PV derivative (LAC and LACΔ*pvl*, respectively), both kindly provided by Frank DeLeo. They are described in detail elsewhere and have been used in murine models of skin infection and pneumonia [11], [13], [19] and in a rabbit model of bacteremia [12]. PVL production by the LAC strain was checked by using a specific ELISA method [21] in the supernatants of inoculum samples.

### Preparation of bacterial inocula

The organisms were stored at −80°C until use. Prior to experiments they were cultured in casein hydrolysate and yeast extract medium (CCY) at 37°C for 18 h with shaking. After centrifugation the supernatants were passed through 0.22-µm filters and stored at −20°C until PVL quantification by ELISA. The pellets were washed and resuspended in phosphate-buffered saline solution (PBS) to the desired bacterial density immediately prior to inoculation. All inocula were quantified by plating serial dilutions on tryptic soy agar (bioMérieux- France).

### Experimental model

Norden's method [22] was used to induce osteomyelitis in female New Zealand white rabbits weighing between 2 and 3 kg. The rabbits were housed in individual cages and received food and water *ad libitum*. The experimental protocol complied with French legislation on animal experimentation and was approved by the Animal Use Committee of Maison Alfort Veterinary School. The animals were anesthetized by intramuscular injection of 25 mg/kg ketamine (Vibrac France) and 25 mg/kg Xylazine Rompum® 2% (Bayer Santé, Division Santé Animal, Puteaux, France). Before *S. aureus* challenge (on day 0), 500 µl of venous blood was drawn and serum was stored at −20°C. An 18-gauge needle was inserted percutaneously through the lateral aspect of the right tibial metaphysis into the medullary cavity. Infection was induced by direct injection of sclerosing agent (0.1 ml of 3% sodium tetradecyl sulphate (Trombovar®)), followed by 0.2 ml of inoculum and 0.1 ml of saline. Patch analgesia (Durogesic®) was given for 7 days following surgery.

Animals were assigned to receive a low inoculum (8×10^5^ CFU) or a high inoculum (4×10^8^ CFU) of LAC or LACΔ*pvl* in order to detect a possible inoculum effect on PVL expression. These inocula were selected on the basis of pilot experiments designed to determine the dose necessary to induce persistent infection with each strain in more than 85% of animals 28 days after inoculation. LAC and LACΔpvl challenge was always performed simultaneously in order to minimize the influence of experimental conditions. Since a chromosomally-restored derivative of the LACΔ*pvl* was not available, no complementation group was included in the experiment.

### Macroscopic aspect and bacterial density of bone

The animals were monitored daily for general and local signs of infection (mobility, aspect of the legs) and were weighed weekly. Moribund animals (immobile, unable to be aroused from a recumbent position, and unable to access food and water) were euthanized by rapid intravenous injection of pentobarbital [23].

Animals were killed 7 days (D7) or 28 days (D28) after infection in order to assess the impact of PVL on the time course of osteomyelitis. Before sacrifice, venous blood was drawn for blood culture and serum samples (≥500 µl) were stored at −20°C for later determination of anti-PVL antibody and C-reactive protein titers. The right leg was visually examined, the tibia and femur were dissected out from the surrounding soft tissues, and the knee joint space, soft tissues, compact bone and medulla were grossly inspected. The following variables were evaluated: the presence and location of purulent exudates in the joint space; soft tissue and bone abscesses; the severity of tibial metaphysis deformation; and spread of the infection to the tibial diaphysis. The macroscopic aspect was noted and photographed. Several evaluations were made on photographs, by an investigator who was unaware of the group attribution. The blinded evaluation was reproducible and consistent with autopsy findings.

The upper third of the tibia was frozen in liquid nitrogen, crushed in a pulverizer (Spex 6700; Freezer/Mill Industries Metechen, NJ), suspended in 10 ml of sterile saline, and quantitatively cultured on tryptic soy agar. Samples of pulverized tibia and subcutaneous abscess fluid were also stored at −20°C for microbiological studies of bone isolates.

### Imaging studies and histopathological examination

Serial MRI was performed on 6 rabbits infected with the low inoculum (3 with LAC and 3 with LACΔ*pvl*) at 7, 14 and 21 days post-infection. These rabbits were killed 28 days post-infection for macroscopic examination and tibial bacterial counts.

MRI, coupled with plain radiography and histopathological examination of the infected legs, was also performed on two animals injected with Thrombovar® alone, 6 animals challenged with the high inocula and killed 7 days post-infection (3 with LAC and 3 with LACΔ*pvl*) and 8 animals killed 28 days post-infection (4 with LAC and 4 with LACΔ*pvl*). In these rabbits, muscle and joint involvement was recorded on the basis of gross signs and histological examination.

MR imaging was performed with a 1.5-Tesla device (Intera; Philips Medical System, Eindhoven 5600 PB, Netherlands) equipped with a surface coil (Sense-Flex-S). The anaesthetized animal (Ketamine and Xylazine Rompum® 2%) was placed on its back with the lower limbs extended. The MR imaging protocol included several classical sequences (T1, T2, PD SPIR, STIR, and 3D WatSF), using a 140-mm field of view. The 3D WatSF sequence (TR: 20 ms, TE: 7.5 ms, flip angle of 50°, matrix 166×512, section thickness 1.5 mm, every 0.8 mm) yielded acceptable reformatted images in the frontal or sagittal plane of the tibia. Moreover, this water-selective sequence was most sensitive for changes due to the infection, the replacement of yellow (lipid) marrow by water representing the earliest abnormality. Images were acquired and examined by a radiologist who was unaware of the group attribution.

For histopathological studies, the right leg, including half the femur, the entire tibia and the foot, was excised. The skin was removed and the leg was fixed for 48 hours in phosphate-buffered 10% formaldehyde. The tibia and femur were then decalcified in 10% nitric acid for 48–72 hours. A perpendicular section was cut through the knee and patella and was used to prepare serial macroscopic slices including the knee, the entire tibia and the distal femur. These slices were dehydrated in alcohol and embedded in paraffin. Histological sections 4 µm thick were then cut and stained with hemalun-eosine-safran (HES). Some samples were also Gram stained. The slides were examined by a pathologist who was unaware of the group attribution. The following parameters were evaluated for each joint, bone and muscle specimen: 1) inflammation (presence, stage, main inflammatory cells), 2) other pathological changes, such as fibrosis, edema, necrosis, and abscesses; and 3) presence of bacteria.

### Serum antibody assay

Antibodies against PVL (before *S. aureus* challenge and at sacrifice) were measured with a specific ELISA method. Specific anti-LukS-PV antibodies were quantified with a protocol adapted from Croze et al [24], with a peroxidase-conjugated swine anti-rabbit polyvalent IgG diluted 1∶1000 (DAKO). Serial dilutions of anti-luk-S polyclonal rabbit serum (bioMérieux) were used for calibration. The results were expressed in arbitrary units per milliliter (AU/mL), one arbitrary unit corresponding to the amount of anti-LukS-PV antibodies contained in a 1/107 dilution of the polyclonal rabbit reference serum.

### C-reactive protein (CRP) assay

CRP was assayed in serum by using a specific ELISA method, as recommended by the provider (ALPCO).

### Statistical analysis

Percentages (of infected animals, bone marrow involvement, bone deformation, and muscle or joint involvement) were compared with Fisher's exact test. The non parametric Mann-Whitney U test was used to compare tibial bacterial counts and anti-PVL antibody and CRP titers. P values <0.05 were considered to denote significant differences.

## Results

### Selection of inoculum sizes

Preliminary studies were performed with three inocula, of 8×10^5^ CFU, 4×10^6^ CFU and 4×10^8^ CFU. Six animals challenged with each inoculum were killed on D7 and D28. The low inoculum of LAC and the high inoculum of LACΔ*pvl* induced persistent infection on D28 in respectively 90% and 86% of animals, and were therefore used in the rest of the study.

### Characteristics of osteomyelitis induced by the low inocula

All rabbits challenged with the low inoculum of LAC (n = 19) or LACΔ*pvl* (n = 19), including the animals used in the preliminary inoculum-ranging experiments, were used for macroscopic and microbiological evaluation. One animal each in the LAC and LACΔ*pvl* groups died prematurely, on D4 and D6 respectively. Nine rabbits in each group were sacrificed on D7 and on D28.

Mean body weight changed significantly on D7 in the LACΔ*pvl* group (+7.5%, *P* = 0.025 vs D0) but not in the LAC group (+3.5%, *P* = 0.31 vs D0). Significant weight gain was observed in the two groups on D28 (+29% and +34% in the LAC and LACΔ*pvl* groups, respectively; *P*<0.001 vs D0 in both groups)

**Figure 1:**
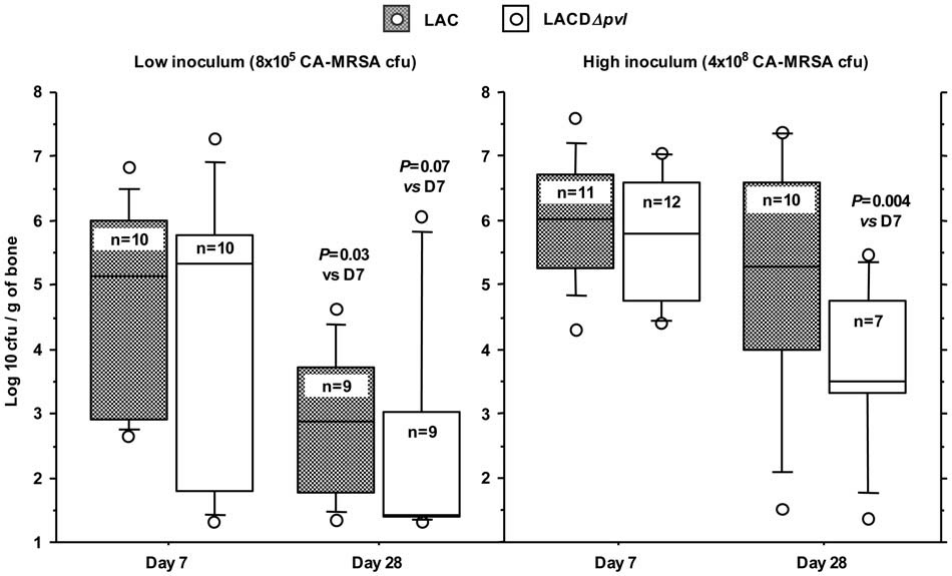
Bacterial counts in rabbit tibia. Box plot representation (10, 25, 50, 75, 90th percentiles and outliers) of bacterial counts in rabbit tibia 7 days (D7) and 28 days (D28) after inoculation with 8×10^5^ (low) or 4×10^8^ (high) CFU of LAC and LAC Δ*pvl* CA-MRSA. *P* values for differences between D28 and D7 were obtained with the non parametric Mann-Whitney U-test.

Combined analysis of the D7 and D28 results showed that significantly more rabbits challenged with LAC than with LACΔ*pvl* had infected bone (18/19 vs 12/19, *P* = 0.042), suggesting that PVL enabled better colonization by and/or survival of the parental strain LAC. However, bacterial counts in crushed bone were similar in the two groups (Figure 1). Bacterial counts tended to be lower on D28 than on D7 (Figure 1).

Macroscopically, the infection mainly involved bone marrow which was yellow/white in all infected animals and necrotic in two animals in each group. Bone deformation was observed in only 4/38 animals overall (3/19 and 1/19 in the LAC and LACΔ*pvl* groups, respectively; *P* = 0.60) and muscle abscesses were present in only 2/38 animals (2/19 and 0/19 respectively; *P* = 0.49).

**Figure 2:**
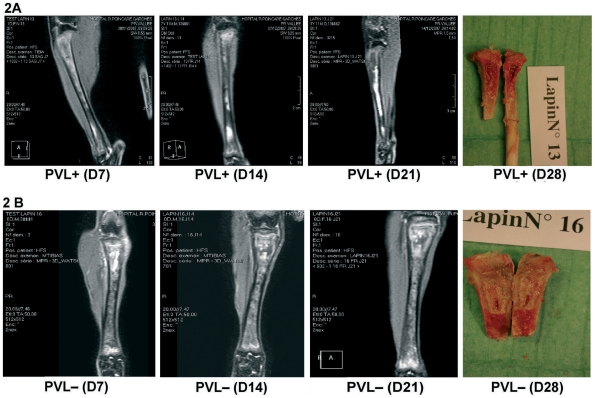
Serial MRI of a rabbit challenged with a low inoculum of LAC (PVL+) or LACΔ*pvl* (PVL−). 2A - Serial magnetic resonance imaging (MRI) of a rabbit infected with a low inoculum of LAC (PVL+); the mean tibial bacterial count was 4.64 Log10 CFU/g of bone on D28. The infection appears as a marrow hypersignal on D7, that becomes more intense and extends to the entire diaphysis on days 14 and 21. A medullary abscess was found at autopsy on D28. 2B - Serial MRI of a rabbit challenged with a low inoculum of LACΔ*pvl* (PVL−). An intramedullary abscess was visible in the metaphysis and proximal diaphysis on D7, appearing as an intense marrow hypersignal. Central necrosis was noted on images obtained on D14 and D21. Bone marrow necrosis was observed macroscopically on D28. The mean tibial bacterial count on D28 was 6.08 Log10 CFU/g of bone.

Serial MRI studies, performed on 3 animals in each group, systematically showed signs of infection on *all but the T1 sequences*, with a marrow hypersignal initially restricted largely to the metaphysis; it gradually became more intense in 3 rabbits (2 LAC, 1 LACΔ*pvl*) (Figure 2A), remained stable in 2 rabbits (1 LAC, 1 LACΔ*pvl*), and was already severe on D7 (extending to the diaphysis) in 1 rabbit (1 LACΔ*pvl*, Figure 2B). Thus, both the PVL-positive strain and its PVL-negative derivative caused severe bone marrow involvement.

### Characteristics of osteomyelitis induced by the high inoculum

Respectively 29 and 26 rabbits received the high inocula of LAC and LACΔ*pvl*. Six animals (4 LAC and 2 LACΔ*pvl*) died or were euthanatizied prematurely. Respectively 11 and 12 rabbits infected with LAC and LACΔ*pvl* were killed on D7, and 7 and 5 rabbits were killed on day 28 for microbiological evaluation and macroscopic inspection.

Macroscopic findings were also recorded in the 14 rabbits (7 in each group) used for imaging and histopathological studies.

On D7, body weight loss was significant and similar in the two groups (average −8% with LAC and −10% with LACΔ*pvl*; *P* = 0.014 and *P*<0.0001 vs D0, respectively), reflecting more severe disease than that observed with the low inocula. Body weight returned to baseline by D28, with similar weight recovery in the two groups.

All the animals had infected bones on D7, and the infection persisted on D28 in 90% and 86% of animals challenged with LAC and LACΔ*pvl*, respectively. As with the low inoculum, mean bacterial counts were lower on D28 than on D7 (Figure 1). However, the decline was significant in rabbits challenged with LACΔ*pvl* but not in those challenged with LAC, again suggesting that PVL enhanced bacterial survival.

**Figure 3:**
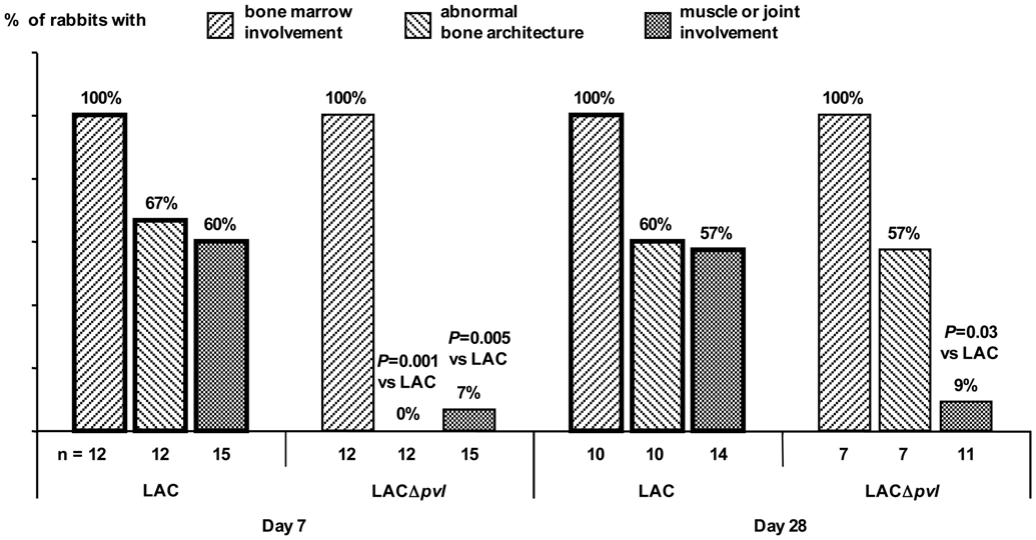
Bone and muscle/joint involvement after challenge with a high inoculum of LAC and LAC Δ*pvl*. Percentages of rabbits with bone marrow involvement, bone deformation and muscle/joint involvement 7 (D7) and 28 (D28) days after challenge with a high inoculum of LAC and LAC Δ*pvl*. n is the total number of animals. *P* values for differences between the c and LAC groups were obtained with the non parametric Mann-Whitney U-test.

Osteomyelitis induced by the high inocula was associated with severe local macroscopic changes at autopsy, with bone marrow involvement, bone deformation, and extension to the muscle (localized abscesses, myositis, sinus tract) and joint (purulent arthritis) in some rabbits (Figure 3, Table 1). Bone deformation and muscle/joint involvement were seen in respectively 67% and 60% of LAC-infected rabbits on D7, compared to respectively 0% and 7% of LACΔ*pvl*-infected rabbits (*P* = 0.001 and *P* = 0.005 respectively) (Figure 3). On D28 the frequency of bone deformation was similar in the two groups (∼60%), while muscle/joint involvement was still significantly more frequent with LAC than with LACΔ*pvl* (57% versus 9%, *P* = 0.033). Thus, PVL+ osteomyelitis was an acute infection with rapid local extension, contrary to PVL− osteomyelitis.

**Table 1:** Macroscopic findings in rabbits challenged with a high inoculum: details of soft tissue and joint changes.

Strain	Days	Purulent arthritis	Abscess	Myositis	Sinus tract
**LAC**	**D7**	1/15	6/15	0/15	2/15
	**D28**	4/14	2/14*	3**/14	2/14
**LAC Δ ** ***pvl***	**D7**	0/15	1/15	0/15	0/15
	**D28**	1/11	0/11	0/11	0/11

Results are expressed as number of rabbits with muscle/joint involvement on total number of rabbits challenged with LAC or LAC Δ *pvl*.

* including one rabbit with concomitant purulent arthritis.

** including two rabbits with concomitant purulent arthritis.

**Figure 4:**
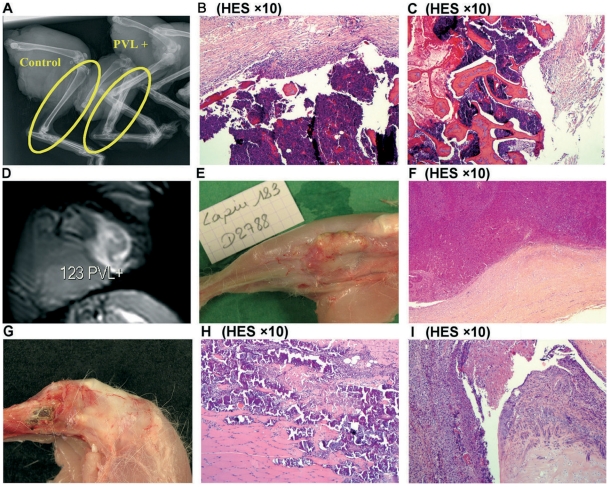
Typical radiological and histological findings after challenge with a high inoculum of LAC (PVL+). A, B, C: Radiological and histological findings 28 days after challenge with a high inoculum of LAC (PVL+). Note the major deformation and widening of the entire diaphysis, compared to the control animal injected with sclerosing agent alone. Histological studies showed an intramedullary abscess and signs of chronic osteomyelitis. B: Bone abscess: the bone marrow space is filled with altered neutrophilic PMN, accompanying bone destruction and necrosis. C: Sequestrum: area of necrotic bone surrounded by an acute inflammatory exudate (pus). D, E, F: 28 days after challenge with a high inoculum of LAC (PVL+), note the soft tissue abscess surrounded by a fibrous layer on MRI and histological studies. G, H, I: Histological findings in a rabbit (PVL+) that died on D8 after challenged with a high inoculum of LAC. Histological sections (H) show muscle involvement with diffuse necrosis and dystrophic calcification, and (I) a purulent exudate in the joint cavity.

MRI and plain radiography, performed on 7 LAC-infected and 7 LACΔ*pvl*-infected rabbits, showed the same signal modifications as in the rabbits challenged with the low inocula. However, imaging of isolated legs, used for the high-inoculum group, was less informative than imaging of live animals (used for the low-inoculum group), thus explaining why no modifications were seen on Day 7 in the high-inoculum group. Radiographic changes were apparent on D28 in half the rabbits infected with LAC (2/4) and LACΔ*pvl* (2/4), with widening and deformation of the tibial metaphysis and diaphysis, ostelysis, and subperiosteal osteogenesis. Typical radiological and histological aspects in infected rabbits, and their correspondence with macroscopic findings, are shown in Figure 4.

### Anti-PVL antibody titers

**Figure 5:**
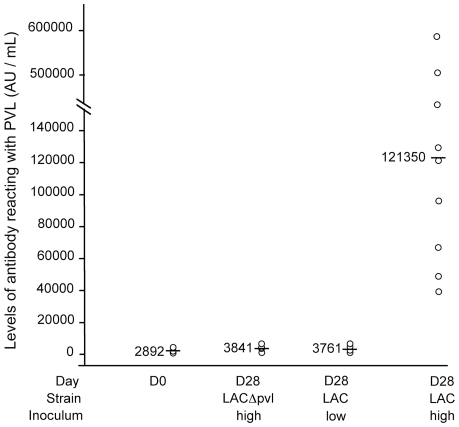
Anti-PVL antibody levels in sera of rabbits. Distribution of anti-PVL antibody levels in 24 sera obtained before *S. aureus* challenge (D0); 7 sera obtained 28 days after challenge with LACΔ*pvl*, 9 sera obtained 28 days after challenge with a high inoculum of LAC, and 8 sera obtained 28 days after challenge with a low inoculum of LAC. Bars show the median values. The median anti-PVL titer increased 31-fold on D28 versus D0 after challenge with a high inoculum of LAC (p<0.001). No significant change was detected at D28 versus D0 in animals challenged with LACΔ*pvl* or with the low inoculum of LAC.

Anti-PVL antibodies were measured in blood drawn before infection (D0) and at the time of sacrifice (D28) (Figure 5). The median anti-PVL titer in rabbits infected with the high inoculum of the LAC strain increased 31-fold on D28 (range 39 190–613 100 AU/mL, median 121 350 AU/mL) versus D0 (range 1 270–4 220 AU/mL, median 3 129 AU/mL). No significant change in the anti-PVL titer was detected on D28 after challenge with LACΔ*pvl*, at either the high inoculum (D0 range 1 490–4 330 AU/mL, median 3 620 AU/mL; D28 range 1 490–6 894 AU/mL, median 3 841 AU/mL) or the low inoculum (D0 range 1 215–3 870 AU/mL, median 2 388 AU/mL; D28 range 1 284–16 290 AU/mL, median 2 905 AU/mL), or with the low inoculum of the LAC parent strain (D0 range 1 233–5 164 AU/mL, median 2 852 AU/mL; D28 range 1 391–18 650 AU/mL, median 3 761 AU/mL).

### PVL production in vitro and in vivo

PVL production by strains recovered from the inocula and infected bones was measured in vitro and remained constant, ranging from 7.5 to 10 µg/ml LukS-PV. The PVL concentration was also measured in some pus samples. As much as 0.65 µg/ml of LukS-PV was detected in the pus of a knee abscess in a LAC strain-infected animal.

### CRP

**Figure 6:**
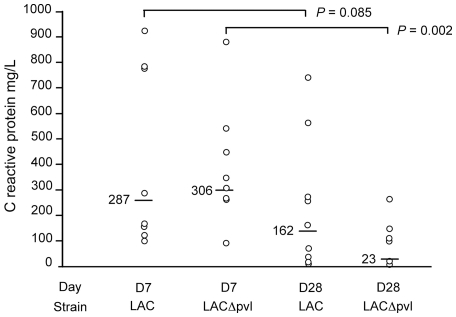
C-reactive protein (CRP) values in rabbits. Distribution of C-reactive protein (CRP) values in 36 rabbits (9 samples obtained on D7 from rabbits infected with LAC, 9 samples obtained on D28 from rabbits infected with LACΔ*pvl*, 9 samples obtained on D28 from rabbits infected with LAC, and 9 samples obtained on D28 from rabbits infected with LACΔ*pvl* (all animals challenged with the high inoculum). Bars show the median values. The fall in the median CRP values from D7 to D28 was statistically significant for LACΔ*pvl* infection (p = 002), but not for LAC infection (p = .085).

C-reactive protein levels were measured on D7 and D28 after challenge with the high inocula of LAC and LACΔ*pvl*. CRP levels were high in both groups on D7 (LAC: 100.6–924.0 mg/L, median 287.0 mg/L; LACΔ*pvl*: 91.3–879.9 mg/L, median 306.8 mg/L), and fell on D28, significantly with LACΔ*pvl* (p = 0.002) but non significantly with LAC (p = 0.085) (figure 6).

## Discussion

Hematogeneous osteomyelitis has long been recognized as a major clinical syndrome of *S. aureus* disease in children. Since the antibiotic era, acute osteomyelitis has become a less frequent cause of hospital admission and death [15]. Concomitantly with the recent worrying emergence of CA-MRSA strains, the incidence of *S. aureus* osteomyelitis, which represents 7% of infections due to staphylococci in hospitalized children in the United States, doubled between 2002 and 2007 [9].

Panton and Valentine themselves suspected a role of PVL in osteomyelitis [25], reporting that the leukocidin was produced in large amounts by staphylococcal strains causing severe infections [16]. Yet no experimental studies of the impact of PVL on the course of osteomyelitis have been conducted to date. Here we provide evidence that PVL plays a role in rabbit CA-MRSA osteomyelitis, i) by enhancing the persistence of the infection, even with a low inoculum, and ii) by facilitating local extension during the early phase of infection after challenge with a high inoculum.

More rabbits had infected bones on D7 and D28 after challenge with a low inoculum of LAC than with a low inoculum of the PVL-negative isogenic derivative, suggesting that PVL enhances bacterial persistence at the site of infection. PVL had a similar but less marked influence after challenge with the high inoculum. Our findings are consistent with the recent report by Diep et al, which suggests that PVL plays a role in the early stages of rabbit CA-MRSA bacteremia by enhancing the survival and spread of the strain USA300 [12].

PVL-positive osteomyelitis induced by the high inoculum caused bone deformation and muscle/joint involvement in more than 60% of rabbits, as early as 7 days post-infection. In contrast, the PVL-negative isogenic derivative failed to cause bone deformation or muscle involvement on D7, while muscle involvement was infrequent and less severe 28 days post-infection than with the parent strain.

A determining role of PVL in muscle extension has been also observed in a mouse model of skin infection: severe myositis was observed in mice inoculated subcutaneously with LAC but not in those inoculated with LACΔ*pvl* [19]. These experimental findings are consistent with the more severe local disease observed in children and young adults with osteomyelitis due to PVL-producing strains. Bocchini et al [18] have shown that patients infected with PVL+ *S. aureus* isolates are significantly more likely to have concomitant myositis or pyomyositis than are patients with PVL− *S. aureus* isolates. Dohin et al [5] showed that patients with PVL+ infection were more likely to have abnormalities on initial radiographic examination, a finding suggesting a more aggressive course.

PVL targets immune cells such as polymorphonuclear neutrophils (PMNs), monocytes and macrophages. Depending on the concentration, PVL-induced pores cause cytokine release and cell death by apoptosis or necrosis, thus enhancing the inflammatory response and causing extensive tissue necrosis [26]. The leukotoxic activity of PVL could thus explain the differences in soft tissue involvement observed here between the PVL+ and PVL− strains. The inflammatory response to *S. aureus* also seems to be enhanced by PVL in the clinical setting, with a higher erythrocyte sedimentation rate (ESR) and a higher C-reactive protein level [5]. We found no difference in the day-7 CRP level between animals infected with LAC and LACΔ*pvl*. In contrast, the CRP level fell significantly on D28 in animals infected with LACΔ*pvl* but not in those infected with the PVL-positive parent strain LAC. This fall in the CRP level in LACΔ*pvl*-infected rabbits coincided with a decline in bacterial counts in bone. Conversely, the persistently high CRP levels in LAC-infected rabbits were associated with severe extra-osseous involvement and were possibly related to the proinflammatory properties of PVL.

Anti-PVL antibody titers rose markedly in rabbits challenged with the high inoculum of the LAC strain. This in keeping with reports that, compared to children with soft-tissue infections, children with invasive *S. aureus* infections (including pneumonia and bone and joint infections) develop dominant anti-LukF and -LukS responses that correlate with markers of inflammation [27].

As virulence gene expression depends on the location and the stage of infection [14], the use of an appropriate experimental model is crucial to understand the impact of PVL in human bone and joint infections. The model used here resembles pediatric hematogenous osteomyelitis in several respects [28]. First, the infection begins in the tibial metaphysis and progresses to diffuse osteomyelitis. As in humans, it becomes chronic if left untreated [15]. MRI signs can be detected as early as day 5 after inoculation [29], followed by radiographic changes [22]. In contrast, unlike acute osteomyelitis in children, this model involves local inoculation and requires the use of a sclerosing agent to create small-vessel thrombosis and micronecrosis, thereby enhancing bacterial proliferation [30]. Attempts to induce experimental osteomyelitis reproducibly without the use of a sclerosing agent or trauma have been unsuccessful [28], [31]. In preliminary experiments we inoculated rabbits with 4×10^6^ CFU of the LAC strain, either alone or concomitantly with Thrombovar®, and found that the bone was sterile on D7 in respectively 3/5 and 0/6 animals.

The inoculum required to provoke osteomyelitis in humans is not known, and could depend on both bacterial and host factors. As in most experimental infections, the inoculum necessary to induce osteomyelitis in rabbits may be higher than in humans. Our use of two different inocula (low and high) and two time points (D7 and D28) contributed to unmasking the role of PVL. Osteomyelitis induced by the high inoculum closely mimicked severe pediatric cases of PVL+ *S. aureus* infection described by Bocchini et al [18] (presence of myositis and pyomyositis) and by Dohin et al [5] (early cortical bone deformation).

In addition, the choice of adequate experimental readouts is critical. In our study, bacterial counts in bone – the classical parameter used in therapeutic studies but one that is never determined in human infections – were similar with the two strains. In contrast, macroscopic findings, which are also used to evaluate the severity of human osteomyelitis, were strain-dependent and correlated closely with radiological and histopathological findings.

In conclusion, our findings, obtained in a rabbit model of osteomyelitis due to a PVL-expressing strain of *S. aureus* and its isogenic PVL-negative derivative, supports the role of PVL as a virulence factor that enhances bacterial persistence and local spread. This is consistent with clinical reports of severe ostemyelitis due to PVL-producing *S. aureus* strains in children. In addition to clarifying the pathophysiologic role of PVL in osteomyelitis, our experimental model should be suitable for assessing new treatments.

The erosion of the antibiotic armamentarium has prompted a search for other ways to prevent and treat serious staphylococcal infections. Our results call for the evaluation of new therapies to combat PVL such as anti-PVL intravenous immunoglobulin.
